# Nuclear Receptors and Clearance of Apoptotic Cells: Stimulating the Macrophage’s Appetite

**DOI:** 10.3389/fimmu.2014.00211

**Published:** 2014-05-12

**Authors:** Noelia A-Gonzalez, Andrés Hidalgo

**Affiliations:** ^1^Department of Epidemiology, Atherothrombosis and Imaging, Fundación Centro Nacional de Investigaciones Cardiovasculares, Madrid, Spain

**Keywords:** macrophages, nuclear receptors, liver X receptors, apoptotic cell clearance, inflammation

## Abstract

Clearance of apoptotic cells by macrophages occurs as a coordinated process to ensure tissue homeostasis. Macrophages play a dual role in this process; first, a rapid and efficient phagocytosis of the dying cells is needed to eliminate uncleared corpses that can promote inflammation. Second, after engulfment, macrophages exhibit an anti-inflammatory phenotype, to avoid unwanted immune reactions against cell components. Several nuclear receptors, including liver X receptor and proliferator-activated receptor, have been linked to these two important features of macrophages during apoptotic cell clearance. This review outlines the emerging implications of nuclear receptors in the response of macrophages to cell clearance. These include activation of genes implicated in metabolism, to process the additional cellular content provided by the engulfed cells, as well as inflammatory genes, to maintain apoptotic cell clearance as an “immunologically silent” process. Remarkably, genes encoding receptors for the so-called “eat-me” signals are also regulated by activated nuclear receptors after phagocytosis of apoptotic cells, thus enhancing the efficiency of macrophages to clear dead cells.

## Apoptotic Cell Recognition and Clearance

Macrophages are professional phagocytes that clear unwanted cells both in the steady-state and during the resolution phase of the immune response. Phagocytosis of apoptotic cells is crucial for development and reproduction. It is also important for the regulation of the immune system because, unlike other phagocytic processes such as phagocytosis of necrotic cells or bacteria, clearance of apoptotic cells does not lead to a pro-inflammatory response in macrophages (1). Apoptotic cell clearance occurs in four steps: sensing of the apoptotic cell, recognition, engulfment of the corpse, and processing of the engulfed material (2). In the last few years, many novel molecules and signaling pathways have been described as key regulators of these steps. In the first step, recognition of the target cell occurs via the so-called “find-me” signals that are released by the apoptotic cell and promote the migration of the phagocyte (3). Examples of these soluble “find-me” signals are the nucleotides ATP and UTP (4), fraktalkine (CX3CL1) (5), and lysophosphatidylcholine (LPC) (6). Apoptotic cells exhibit “eat-me” signals in their surface that are recognized by the phagocyte, either directly or through bridging molecules. The best described “eat-me” signal is phosphatidylserine (PtdSer) exposed in the outer leaflet of the membrane of apoptotic cells (7). Scavenger receptors such as CD36; tyrosine kinases, such as Mertk; or immunoglobulin and mucine domain-containing molecules, such as TIM-4, are membrane receptors that recognize PtdSer (8–10). This recognition may be direct or through soluble factors, such as MFG-E8, Gas6, ProteinS, or the C1q opsonin. The signaling pathways triggered during engulfment then lead to reorganization of the cytoskeleton, and promote internalization of the dying cell (2).

In order to maintain homeostasis, the engulfed material needs to be processed by the phagocyte. When apoptosis occurs, the number of dying cells is typically higher than the number of phagocytes present in the tissue. This disproportion is evident during the resolution phase of inflammation, during the negative selection in the thymus or during germinal center reactions (9, 11–14). However, in all these cases very few if any apoptotic cells can be detected because tissue-resident and recruited macrophages are extremely efficient at clearing up all dying cells, and efficiently processing the extra cargo ingested to prevent the generation of an inflammatory response. This processing entails production of anti-inflammatory cytokines, such as IL-10 and TGF-β1, which are important to initiate the resolution phase or to maintain the process immunologically silent (1, 15, 16). In support of this concept, deficiency in the phagocytosis of apoptotic cells is one of the hallmarks of patients with systemic lupus erythematosus (17). However, the transcriptional regulators of the inflammatory routes triggered by apoptotic cell clearance have only recently begun to be elucidated.

In order to maintain a normal metabolic rate the engulfing phagocyte must process the extra metabolites provided by the ingested apoptotic cells, as excessive metabolite accumulation may be noxious. Cholesterol efflux is induced in phagocytes by apoptotic cells exposure, and is dependent on phosphatidylserine recognition (18). Expression of genes implicated in cholesterol efflux, such as ATP-binding cassette (ABC) transporter genes, is further up-regulated via activation of nuclear receptors (15, 18–20). This metabolic response is thought to maintain cholesterol levels within the phagocyte. However, macrophages generally ingest more than one apoptotic cell and phagocytosis further enhances recognition and engulfment of apoptotic targets (15, 19). Thus, the extra load of cellular components within the phagocyte might also have energetic benefits for the cell, as it needs energy to continue phagocytizing more cells. Park and collaborators defined an inverse relationship between mitochondrial membrane potential and phagocytosis, in which macrophages with low mitochondrial membrane potential are prone to engulf apoptotic cells. The authors showed that the mitochondrial membrane potential increases in the phagocyte after engulfment of apoptotic cells, to later return to baseline levels. Restoration of baseline potentials is ensured by Ucp2, a mitochondrial membrane protein whose levels also increase after engulfment. Ucp2 therefore acts as a “sensor” for phagocytosis that, by maintaining the mitochondrial membrane potential at basal levels, allows continued phagocytosis (21).

Nuclear receptors are a superfamily of ligand-activated transcription factors implicated in metabolic and inflammatory pathways (22). Their key roles in macrophage biology led us and others to explore their activity in apoptotic cell clearance. This review discusses the importance of nuclear receptors during the phagocytosis of apoptotic cells. We will emphasize how the processing of apoptotic cells, through regulation of metabolic genes and anti-inflammatory pathways, is essential to maintain homeostasis.

## Nuclear Receptors at the Interface of Metabolism and Immunity

Nuclear receptors share a highly conserved amino-terminal activation domain, a carboxy-terminal ligand binding domain, a zinc-finger DNA-binding domain, and a second activation C-terminal domain (22). Since Mangelsdorf and collaborators first proposed in 1995 a classification of nuclear receptors based on their ligands and DNA-binding modalities (23), several categories have been proposed (24, 25). A simplistic classification of two types of nuclear receptors is described in the Nuclear Receptors Signaling Atlas resource, NURSA (for more detailed information visit NURSA website: www.nursa.org). In the type I category, hormone receptors undergo nuclear translocation upon ligand activation and bind as homodimers to inverted DNA repeat sequences. This category includes estrogen, glucocorticoid, progesterone, mineralocorticoid, and androgen receptors. Type II nuclear receptors are retained in the nucleus and bind as heterodimers with a different nuclear receptor, the retinoid X receptors (RXR), to direct DNA repeats. Thyroid hormone receptor, Liver X Receptors (LXRs), Peroxisome proliferator-activated receptors (PPARs), or Vitamin D receptors (VDRs), among others, fall into this category. Glucocorticoid receptors, LXRs and PPARs have been linked to the phagocytic capacity and phenotypic polarization of macrophages *in vitro* (26–29). However, the mechanism by which gene regulation by these nuclear receptors impacts tissue homeostasis *in vivo* during apoptotic cell clearance is only now starting to be uncovered.

Proliferator-activated receptors are comprised of three isoforms (PPARα, PPARγ, and PPARδ), and are expressed in multiple cell types and tissues. Their endogenous ligands are lipids, such as unsaturated fatty acids, VLDL, and LDL (22). They are essential for fatty acid metabolism by controlling the expression of genes involved in transport, synthesis, activation, and oxidation of fatty acids (30). PPARα activity is mostly restricted to the metabolism of fatty acids, although remarkable immune-regulating properties have been attributed to PPARα due to its capacity to regulate *Cpt1*, a gene involved in T cell function (31).

PPARδ and PPARγ, like other lipid-activated nuclear receptors, are involved in the regulation of inflammatory genes in macrophages. PPARδ is ubiquitously expressed and exhibit pleiotropic functions that range from metabolism, development, and reproduction to inflammation (32). PPARδ represses the expression of inflammatory genes through sequestration of the transcriptional repressor BCL-6 (33). It has been implicated in the phagocytosis of apoptotic cells and prevention of systemic autoimmune diseases (19). Analogous functions in apoptotic cell clearance and autoimmune processes have been described for PPARγ in macrophages (20). Its importance in lipid metabolism is underlined by the variety and function of its target genes, including the scavenger receptor CD36, lipoprotein lipase (LPL), and the nuclear receptor LXRα (34, 35).

LXRα and LXRβ, the two isoforms of LXR, are physiologically activated by oxidized forms of cholesterol. LXRβ is ubiquitously expressed, whereas LXRα is expressed mainly in myeloid cells, intestine, adipose tissue, adrenal glands, and liver. Both isoforms regulate a variety of genes implicated in cholesterol efflux, including the ABC transporters ABCA1 and ABCG1. Accordingly, they have been shown to be important in the prevention of metabolic diseases such as atherosclerosis (22). LXRs can also be pharmacologically activated by potent synthetic agonists that functionally mimic their endogenous ligands.

Elegant studies in the last 10 years have shown that in macrophages previously challenged with inflammatory stimuli, LXRs can act as trans-repressors of pro-inflammatory genes, by binding to other transcription factors and promoting their deactivation (36–39). Thus, like PPARs, LXRs generate cross-talk between inflammation and metabolism. Several studies have now uncovered important roles for these receptors beyond the regulation of inflammatory gene expression and innate immunity. LXRβ has been implicated in the proliferation of T cells, thus influencing adaptive immunity (40). In addition, we have demonstrated that LXRα is essential for the development of two populations of macrophages in the marginal zone of the spleen that are important for immune responses against T cell-independent antigens (41).

The above described pleiotropic functions of PPARs and LXRs position them as excellent candidates to influence macrophage responses during apoptotic cell clearance, in which regulation of metabolic and inflammatory genes is crucial.

## PPARs and Autoimmunity

Initial evidence implicating PPARγ in apoptotic cell clearance was obtained in the context of reactive oxygen species (ROS) production by macrophages (27). In PMA-stimulated macrophages, the production of ROS was attenuated when fed with apoptotic cells. This anti-inflammatory effect was linked to the activity of PPARγ after apoptotic cell clearance. Electrophoretic mobility shift assays revealed transient activation of this nuclear receptor after apoptotic cell recognition (27). Although Mukundan and collaborators later showed that PPARγ transcripts in bone marrow-derived macrophages were not regulated upon phagocytosis (19), several subsequent reports have confirmed a role for PPARγ activation in apoptotic cell clearance (20, 42). In support of the relevance of PPARγ in apoptotic cell phagocytosis by macrophages *in vivo*, mice with conditional deficiency in the receptor in macrophages show a delay in phagocytosis of apoptotic cells and develop autoimmune kidney glomerulopathy (20).

Mice deficient in PPARδ, either globally or restricted to macrophages, also develop a lupus-like autoimmune phenotype characterized by increased levels of autoantibodies in serum and glomerulonephritis (19). This inflammatory phenotype was associated with defective clearance of apoptotic cells by PPARδ-deficient macrophages. Genomic analysis uncovered a number of target genes that were regulated after phagocytosis in a PPARδ-dependent manner. These genes included the C1qb opsonin, which mediates binding of PtdSer to its receptor on the membrane of the phagocyte, and was described as a direct target of PPARδ–RXRα heterodimers. Through this mechanism, phagocytosis is promoted by clearance itself, as double feeding experiments demonstrated that macrophages increased their phagocytic capacity following successive rounds of apoptotic cell feeding (19).

Similarly, RXRα- and PPARγ-deficient macrophages showed impaired apoptotic cell clearance. In addition, engulfment of apoptotic cells failed to down-regulate inflammatory cytokines in LPS-stimulated macrophages derived from RXRα- and PPARγ-deficient mice. As noted above, these mice develop glomerulopathy and proteinuria, both hallmarks of kidney autoimmune disease (20). As with PPARδ, the activity of these nuclear receptors is induced after phagocytosis of apoptotic cells, thereby promoting the transcription of genes encoding membrane receptors and opsonins required for further recognition and engulfment of apoptotic cells. These studies underscore the importance of nuclear receptors in phagocytosis, in part by priming the macrophage for continued engulfment of apoptotic targets.

## LXRs and Apoptotic Cell Clearance: Beyond Macrophage Homeostasis

In human macrophages, LXR activation regulates the expression of LXRα (43) and PPARγ (44), thereby creating a positive feedback loop that enhances the phagocytic capacity of macrophages. However, this is not the only role of LXRs in apoptotic cell clearance in human macrophages. Though not a direct target gene of LXRα, Transglutaminase 2 (*Tgm-2*), which encodes a protein-crosslinking enzyme implicated in the phagocytosis of apoptotic cells (45), is regulated in human macrophages after engulfment of apoptotic targets in an LXRα-dependent manner (29). Together with the activity of PPARs during apoptotic cell clearance, these observations establish LXRs and PPARs as molecules that influence the “appetite” of macrophages.

As described above, LXRs are physiologically activated by oxidized forms of cholesterol and are key regulators of cholesterol metabolism by controlling the expression of genes responsible for cholesterol efflux, such as ABCA1. This raises the question of what is the significance of LXR activation during apoptotic cell clearance. When a macrophage ingests an apoptotic cell, the amount of cellular content within the macrophage significantly increases, and the extra cellular material has to be processed. A potential solution to this dilemma is the up-regulated expression of a gene responsible for cholesterol efflux, *Abca1*, upon engulfment of apoptotic cells (18, 46). LXR activation appears to be required for this upregulation of *Abca1* because, in peritoneal macrophages obtained from mice deficient in both LXR isoforms (LXRαβ−/−), changes in *Abca1* mRNA expression were blunted after apoptotic cell clearance when compared to control macrophages (46). Moreover, Kiss and collaborators demonstrated that *Abca1* expression and cholesterol efflux were induced upon PtdSer recognition by the macrophage, implying that engulfment is dispensable for LXR activation. In support of a role for nuclear receptors in cholesterol processing, LXRs and PPARγ antagonists inhibited upregulation of *Abca1* and cholesterol efflux mediated by apoptotic cell clearance (18).

At the time of these studies, LXR activation had been exclusively linked to the metabolic response of the phagocyte during apoptotic cell clearance. Using LXR knock-out mice we observed an impaired phagocytic capacity in LXR-deficient macrophages both *in vivo* and *in vitro*, and this impairment was associated to the development of autoimmunity in these mice (15). Apoptotic cells promote the expression of a number of genes in macrophages after clearance. Some of these genes are regulated in an LXR-dependent manner, such as genes implicated in cholesterol metabolism, glucose transport, and other genes identified as LXR target genes in other studies. Similarly, the expression of *Mertk*, a membrane receptor for apoptotic cells, was also up-regulated by LXRs during phagocytosis or after activation with synthetic LXR ligands, and was identified in these studies as a novel direct target of LXR (15). Together, the responses triggered by LXR activation contribute to enhancing recognition and further engagement of apoptotic targets as evidenced by the observation that the phagocytic capacity in LXRαβ−/−macrophages does not increase after several rounds of apoptotic cell feeding. Notably, by modulating the expression of inflammation-related genes, LXR also participates in the polarization of the macrophage toward an anti-inflammatory phenotype after engulfment of dying cells. This activity essentially contributes to avoidance of unwanted inflammation, which is illustrated by the lupus-like autoimmune disease developed by LXRαβ−/−mice as they age (15).

Liver X receptors nuclear receptors have more recently emerged as regulators of neutrophil homeostasis (47, 48). Due to their short half-life (estimated in 12.5 h in mice), neutrophils must be efficiently cleared on a daily basis. LXR-deficient mice display neutrophilia in blood and accumulation of neutrophils in the spleen and liver, a phenotype that was accounted for by the impaired capacity of LXR-deficient macrophages to engulf apoptotic neutrophils. Production of IL-23 by macrophages and dendritic cells is a critical signal that controls the levels of neutrophils in blood by acting upstream of IL-17 and G-CSF (49). Importantly, activation of LXRs upon neutrophil engulfment strongly represses IL-23 transcription (47). Extending these studies, we have recently shown that clearance of aged neutrophils in the bone marrow modulates the size and activity of the hematopoietic niche through LXR activation (48). We found that neutrophils cleared from blood enter the bone marrow and are engulfed by macrophages, leading to reductions in the number of niche cells and mobilization of hematopoietic progenitors into the bloodstream. In addition, the transcript levels of LXR target genes in the bone marrow spontaneously increase at the time when neutrophils are cleared in this organ, and mice in which macrophages have been eliminated lacked the normal oscillations in *Abca1* expression. Further, regulation of niche cells and progenitor release are impaired in LXR-deficient mice. Together, these findings uncovered new functions for the homeostatic clearance of dying cells in regulating hematopoietic niches in the bone marrow, and a central role for LXR receptors in coordinating these functions (48).

These recent advances in the field provide examples of the multitude of processes and tissues that are likely regulated by the clearance of apoptotic cells by macrophages, and by the receptors involved in this fundamental process.

## Future Directions

**Figure 1:**
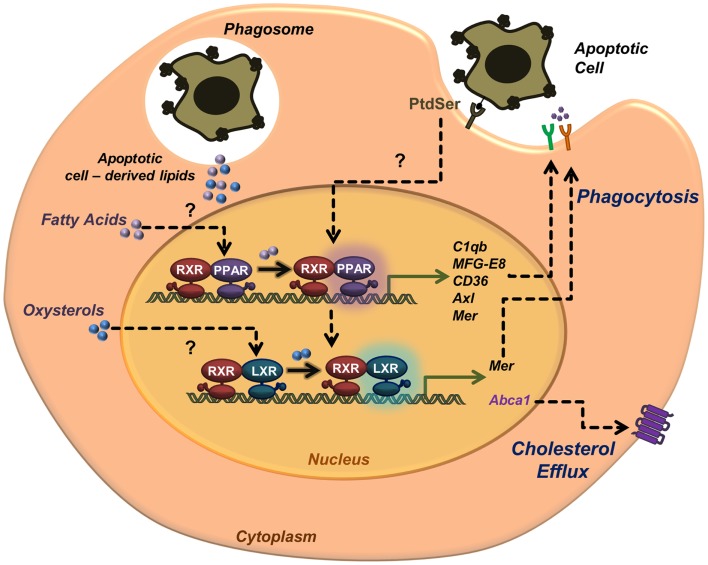
**Activation of nuclear receptors in phagocytes during apoptotic cell clearance**. Apoptotic cell recognition and engulfment promote the transcriptional activity of nuclear receptors LXRs and PPARs. Recognition of phosphatidylserine in the outer leaflet membrane of the apoptotic cell leads to transcriptional activation of ABCA1 and cholesterol efflux. Nuclear receptor activation upon apoptotic cell phagocytosis also leads to upregulation of phagocytic receptors (e.g., Mer, CD36, and Axl) and opsonins (e.g., MFG-E8 and C1qb). Lipids derived from the engulfed apoptotic cells may also serve as source of endogenous ligands to activate PPARs (fatty acids) and LXRs (oxysterols).

Liver X receptors and PPARs are now recognized regulators of the anti-inflammatory response in macrophages after clearance of apoptotic cells. Moreover, these receptors are key players in the recognition and engagement of apoptotic cells by further enhancing phagocytosis through transcriptional regulation of various receptors and bridging molecules. The exact pathways by which LXRs and PPARs are activated during the phagocytosis of apoptotic cells remain an open question in the field (Figure 1). We and others postulated that lipids derived from the engulfed cell might provide ligands for PPARs and LXRs, as demonstrated by the lack of LXR activation when macrophages are fed with sterol-free apoptotic thymocytes (15). Because recognition of PtdSer by macrophages is sufficient to activate an LXR-dependent metabolic program without engulfment (18), additional routes of recognition and engulfment can activate these nuclear receptors.

These novel roles of lipid-activated nuclear receptors in phagocytosis of apoptotic cells raise an interesting issue regarding cell metabolism and bioenergetics. The enhancement of phagocytosis of apoptotic cells mediated by nuclear receptors, might respond to a necessity of generating more energy to continue phagocytizing. Mitochondria provide the majority of the energy supply by oxidative phosphorylation in the respiratory chain. In fact, macrophages with low mitochondrial membrane potential are more prone to phagocyte apoptotic cells (21). Whether nuclear receptors and mitochondria cross-talk during apoptotic cell clearance to enhance phagocytosis arises as an interesting possibility. Supporting this idea, the activity of several nuclear receptors have been defined in mitochondria, regulating gene expression, coordinated with nuclear gene expression, in situations of high energy demand (50). For example, PPARγ co-activator 1α, PGC-1α, collaborates with PPARs to regulate expression of mitochondrial enzymes involved in fatty acid transport and oxidation (51). However, the specific role of nuclear receptors in mitochondrial metabolism during apoptotic cell clearance remains unclear.

An important outcome of this research topic will be the potential therapeutic implications of apoptotic cell clearance in a wide range of inflammatory and metabolic diseases. It has been shown that enhancing engulfment of apoptotic neutrophils *in situ* accelerates the resolution of bacterial infection and lung inflammation (52–54). However, the exogenous administration of apoptotic cells could also lead to autoimmunity, so the therapeutic approaches need to be finely controlled to avoid deleterious effects (55). Targeting nuclear receptors by activation through synthetic ligands, have been proven to ameliorate inflammation in mouse models of autoimmunity (15) and atherosclerosis (56). Though some PPAR agonists have already been approved for clinical use to treat metabolic diseases, a better understanding of nuclear receptor activation during apoptotic cell clearance may pave the way for the development of novel treatments for infectious, inflammatory, and metabolic diseases.

## Conflict of Interest Statement

The authors declare that the research was conducted in the absence of any commercial or financial relationships that could be construed as a potential conflict of interest.
